# Disposal of iron by a mutant form of lipocalin 2

**DOI:** 10.1038/ncomms12973

**Published:** 2016-10-31

**Authors:** Jonathan Barasch, Maria Hollmen, Rong Deng, Eldad A. Hod, Peter B. Rupert, Rebecca J. Abergel, Benjamin E. Allred, Katherine Xu, Shaun F. Darrah, Yared Tekabe, Alan Perlstein, Rebecca Wax, Efrat Bruck, Jacob Stauber, Kaitlyn A. Corbin, Charles Buchen, Vesna Slavkovich, Joseph Graziano, Steven L. Spitalnik, Guanhu Bao, Roland K. Strong, Andong Qiu

**Affiliations:** 1 Columbia University, Russ Berrie Medical Science Pavilion, 1150 Saint Nicholas Avenue, Rm 411, New York, New York 10032, USA; 2 Fred Hutchinson Cancer Research Center, Basic Sciences Division, University of Washington School of Medicine Biochemistry, Immunology, Mail Stop A3-025, Seattle, Washington 98109, USA; 3 Lawrence Berkeley National Laboratory, Chemical Sciences Division, BioActinide Chemistry Group, MS 70A-1150, One Cyclotron Road, Berkeley, California 94720, USA; 4 State Key Laboratory of Tea Plant Biology and Utilization, School of Tea and Food Sciences, Anhui Agricultural University, 130 Changjiang West Road, Hefei 230036, China; 5 Columbia University, New York & Tongji University, School of Life Sciences and Technology, 1239 Siping Road, Shanghai 200092, China

## Abstract

Iron overload damages many organs. Unfortunately, therapeutic iron chelators also have undesired toxicity and may deliver iron to microbes. Here we show that a mutant form (K3Cys) of endogenous lipocalin 2 (LCN2) is filtered by the kidney but can bypass sites of megalin-dependent recapture, resulting in urinary excretion. Because K3Cys maintains recognition of its cognate ligand, the iron siderophore enterochelin, this protein can capture and transport iron even in the acidic conditions of urine. Mutant LCN2 strips iron from transferrin and citrate, and delivers it into the urine. In addition, it removes iron from iron overloaded mice, including models of acquired (iron-dextran or stored red blood cells) and primary (*Hfe*^−/−^) iron overload. In each case, the mutants reduce redox activity typical of non-transferrin-bound iron. In summary, we present a non-toxic strategy for iron chelation and urinary elimination, based on manipulating an endogenous protein:siderophore:iron clearance pathway.

## Abstract (web summary)

Iron overload can be either hereditary or acquired via transfusions, and current treatments include the use of iron chelators that have adverse effects in some patients. Here the authors modify siderocalin to enhance iron excretion in urine, and demonstrate therapeutic efficacy in iron overload mouse models.

Disruption of iron regulation can produce primary, secondary and acquired iron overload disorders. These disorders are characterized by elevated transferrin saturation (>50%), high levels of circulating ferritin (>1,000 μg l^−1^)^1^,^2^,^3^, the emergence of ‘non-transferrin-bound iron' (NTBI) associated with proteins and small organic molecules (NTBI: 0.9–12.8 μmol l^−1^ in thalassemic and 4–16.3 μM in hereditary haemochromatosis sera^2^), and increases in the cellular labile iron pool (LIP)^4^. NTBI and LIP participate in the Haber-Weiss and Fenton reactions which oxidize lipids and proteins and damage DNA by forming hydroxyl, ferryl or perferryl species^5^,^6^. As a result, iron-mediated cell death is found in sensitive organs leading to a variety of human diseases, including liver cirrhosis and hepatocellular carcinoma^7^,^8^, congestive cardiomyopathy^9^, chronic kidney tubular injury^10^, diabetes, hypothyroidism and hypogonadism^11^,^12^,^13^.

The most common form of primary iron overload is hereditary haemochromatosis. Hereditary haemochromatosis is caused by loss of function of genes associated with iron regulation, including *HFE*, *HFE2, HJV*, *HAMP*, *TfR2*, *SLC40A1*, *CP* or *TF*^1^,^3^. Secondary iron overload occurs when iron traffic or iron metabolism is dysregulated by different forms of anaemia (β-thalassemia major, sideroblastic and haemolytic) which can downregulate hepcidin. A third mechanism of iron overload, the syndrome of ‘acquired' iron overload, can be due to the treatment of anaemia by chronic blood transfusions, since each unit of blood contains 200–250 mg of iron which is far in excess of the daily iron loss of 1–2 mg per day^14^. Iron overload from acquired mechanisms also occurs in liver diseases (due to hepatitis C, alcohol)^3^, acute kidney injury (due to hemoglobinuria and myoglobinuria, chemotherapy, ischaemia–reperfusion and transplant ischaemia) and chronic kidney failure^15^,^16^. We previously found excessive iron deposits in the proximal tubules of HIV-associated nephropathy, a form of chronic nephrotic syndrome^17^.

An endogenous system of iron excretion has not been reported in the kidney. Iron is not excreted in the urine because glomerular filtered proteins containing iron are removed from the urinary flow by binding to lumenal membrane cubulin and megalin receptors which direct these proteins to lysosomes. Proximally captured proteins include transferrin, lipocalin 2 (LCN2), lactoferrin, and hepcidin^18^,^19^. Additionally, iron may be reabsorbed through *DMT-1*, *FPN1* and possibly *Zip8* and *Zip14.* Regulation of these processes is now being studied and both circulating and locally expressed hepcidin may play a role in preventing iron loss in the urine^20^. Consistently, hepcidin knockout mice exhibit iron accumulation in the distal nephron and increased urinary iron excretion. Although a complete analysis of iron trafficking in the nephron has not been concluded, it is clear that the kidney can block escape of iron into the urine even in mild forms of iron overload.

Iron overload syndromes can be treated with low molecular weight iron chelators, including the fungal hydroxamate siderophore deferoxamine (DFO, Desferal), the synthetic tridentate deferasirox (Exjade), or the cyclic hydroxypyridinone deferiprone (Ferriprox), some of which can deliver iron into the urine. These agents effectively treat thalassemic iron overload as evidenced by a reduction in tissue iron and an improvement in cardiac function. Yet, they are generally not suitable for prophylactic use because of severe organ toxicity, agranulocytosis^21^,^22^,^23^ and infections^24^ in a small percentage of patients. These untoward effects highlight the need for a new strategy for the non-toxic chelation of iron. Here we propose an alternate strategy for therapeutic removal of excess iron, exploiting an endogenous iron chelation pathway.

We have previously studied an endogenous protein, called LCN2, also known as Neutrophil Gelatinase Associated Lipocalin (NGAL) or Siderocalin (Scn), which has several characteristics useful for the safe excretion of iron. For one, LCN2 is rapidly upregulated when epithelia are exposed to bacteria, hypoxia–ischaemia, or toxic medications, suggesting that it might relieve organ injury^25^,^26^. Second, LCN2 captures catecholates, which in turn bind iron; these are produced by Gram negative bacteria, such as the siderophore Enterochelin (Ent), and potentially by mammals, such as the catechol metabolites of amino acids and dietary polyphenols. Ent and catechol (1,2-dihydroxy benzene) have extremely high affinity for iron (*K*_D_=10^−49^ M and 10^−45.9^ M, respectively)^27^,^28^,^29^ and Ent:Fe^3+^ and catechol:Fe^3+^ bind recognition sites within the calyx of LCN2 with high affinity (Ent:Fe^3+^: *K*_D_=0.4±0.1 nM and catechol:Fe^3+^: *K*_D1_=2.1±0.5 nM, *K*_D2_=0.4±0.2 nM). The ligation of Ent:Fe^3+^ by LCN2 is a critical step in bacteriostasis because sequestration of Ent:Fe^3+^ reduces iron transport to bacteria and limits their growth^30^,^31^,^32^,^33^. The latter distinguishes LCN2:Ent:Fe^3+^ from DFO (*K*_D_=10^−30^ M)^34^, which can deliver iron to *Rhizopus* and induce fatal mucormycosis^24^. Third, the LCN2:Ent:Fe complex is stable under acidic conditions (≥pH4.5), indicating that it should not dissociate in acidified urine. Fourth, ligation of siderophores by LCN2 blocks iron-mediated Fenton reactivity^27^, indicating that LCN2 can safely sequester and transport iron. Fifth, the kidney captures the majority of circulating LCN2 (50–70%) via filtration^35^ followed by reabsorption from the lumen of the proximal tubule, whereupon it is degraded.

These data suggest that the LCN2 pathway might be manipulated to excrete iron into urine and to serve as a novel method of therapeutic iron depletion. To achieve this aim, we generated a mutant form of LCN2, called K3Cys LCN2, which retains high affinity for Ent and for Ent:iron, but can bypass the mechanisms of renal reabsorption, and consequently escape into the urine in a stable complex. K3Cys LCN2 chelates both transferrin-iron and NTBI from the serum of both primary and blood transfusional iron overload mouse models.

## Results

### Generation of LCN2 mutants that can bypass renal reabsorption

**Figure 1:**
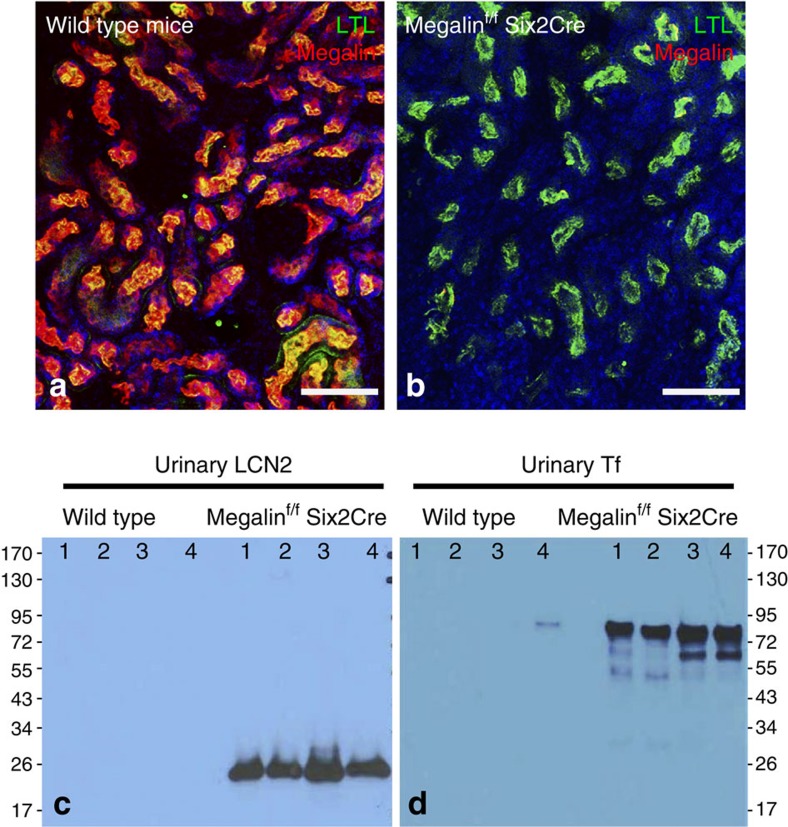
Deletion of megalin results in proteinuria containing LCN2. (**a**) Wild-type mice express megalin (red) in the proximal tubule, which we identified with Lotus-Tetragonolobus-Lectin (LTL, green). Note the nearly quantitative overlap of megalin and LTL (yellow-orange). (**b**) Megalin was deleted with Six2Cre, a driver specific to the nephron. Note LTL stained proximal tubules (green) without staining for megalin (absence of red). (**c**) Megalin deletion resulted in the appearance of urinary LCN2 (detected with mouse specific anti-LCN2) and. (**d**) Urinary Tf. Scale bars **a**,**b**=50 μm.

The capture of LCN2 from the glomerular filtrate is thought to be mediated by megalin, a multi-ligand endocytic receptor positioned at the luminal surface of lotus^+^ proximal tubules (Fig. 1a)^36^. To delete megalin, we crossed megalin-floxed mice^37^ with Six2-CreGFP drivers, resulting in megalin null tubules (Fig. 1b) and the subsequent appearance of proteinuria, containing LCN2 (Fig. 1c). Transferrin (Tf), which is thought to bind to a megalin associated protein called cubilin^38^, was also excreted in the knockout urine (Fig. 1d). These data suggest that megalin restricts protein traffic to the urine.

**Figure 2:**
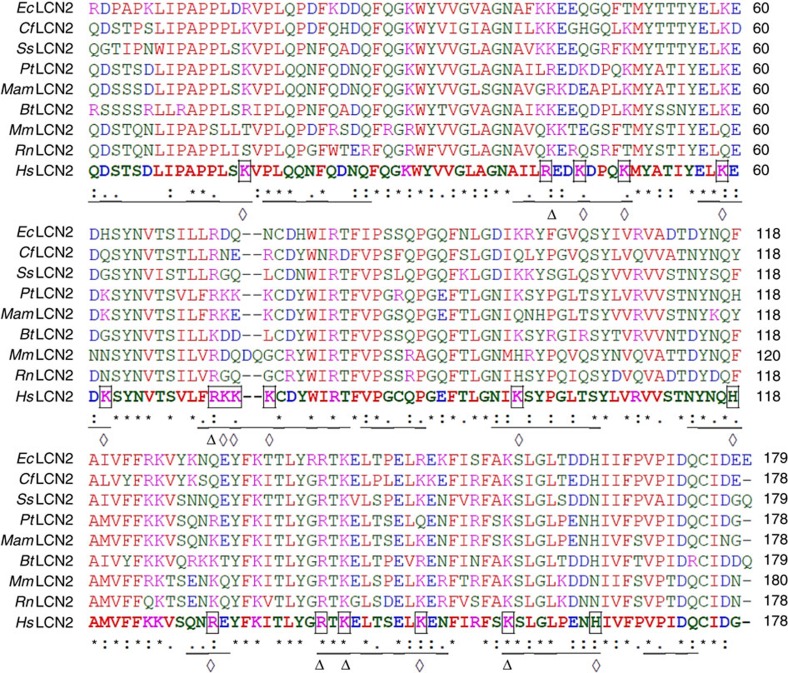
Conservation of surface domains of mammalian LCN2 proteins. LCN2 proteins from horse (*Ec*Lcn2; XP_014591535), dog (*Cf*Lcn2; XP_548441), wild boar (*Ss* Lcn2; NP_001231339), Chimpanzee (*Pt*Lcn2, XP_001153985), Rhesus Monkey (*Mam*Lcn2; XP_001083008), cow (*Bt*Lcn2; XP_605012), mouse (*Mm*Lcn2; NP_032517), rat (*Rn*Lcn2; NP_570097), human (*Hs*Lcn2; NP_005555) were aligned using ClustalW, and surface residues in human LCN2 (Lcn2) underlined. Conserved and the non-conserved surface positively charged residues (Arginine [R], Lysine [K] and Histidine [H]) of *Hs*Ngal are indicated by Δ and ⋄, respectively. Magenta: positive charged residues; Blue: negative charged residues; Red: nonpolar and hydrophobic residues; Green: polar and hydrophilic residues.

It is thought that megalin interacts with its ligands by electrostatic forces between its negatively charged ‘type-A repeats' and positively charged residues of its ligands. Hence, to identify residues required for megalin binding, we mutated 18 positively charged amino acids on the surface of LCN2, based on information from its crystal structure^30^ (K15, 46, 50, 59, 62, 73, 74, 75, 98, 118, 142, 149, 157; H165; and R43, 72, 130, 140). Among these residues, five (K142, 157; R43, 72, 140) were conserved in human, mouse, rat, chimpanzee, cow, dog, pig and Rhesus monkey^31^ and were chosen for site-directed mutagenesis to alanine (Fig. 2); 13 additional positively charged residues in human LCN2 (K15, 46, 50, 59, 62, 73, 74, 75, 97, 149; H118, 165; R130) were not conserved, but nonetheless were mutated to non-positively charged amino acids found at the same structural positions in other mammalian LCN2 proteins. As a result 47 different combinations of LCN2 mutants were generated in BL21 *Escherichia coli* (Supplementary Table 1), then stripped of LPS (∼0.5 EU mg^−1^ protein) and tested for inflammatory responses, which at lower doses were similar to saline (*P*=NS, Student's *t*-test; 0.5–30 mg per mouse; Supplementary Fig. 1).

**Figure 3:**
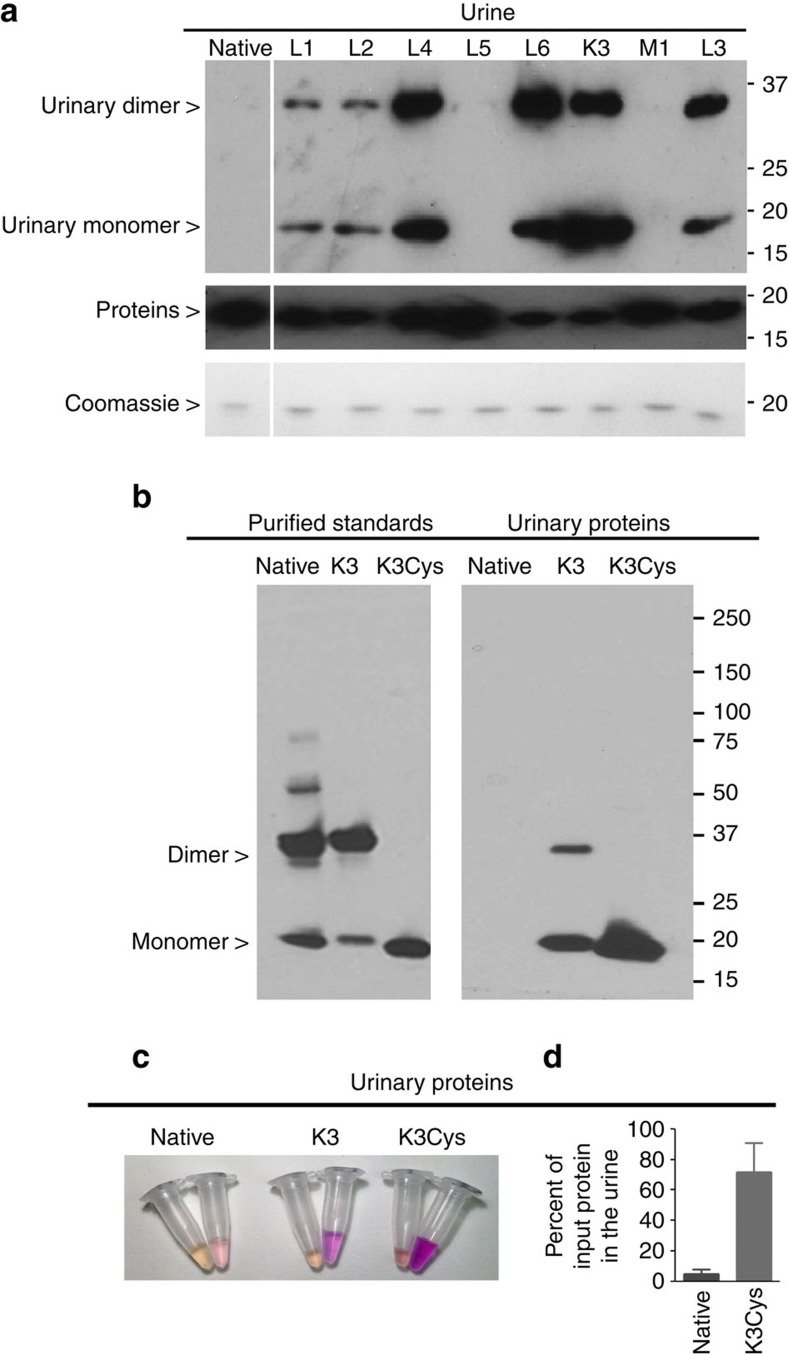
Screening LCN2 mutant proteins for urinary excretion. (**a**) Top panel: mouse urine was collected over 3 h after the inoculation of human LCN2 monomers and dimers of mutant proteins were excreted into the urine, whereas native human LCN2 was not excreted. The urine was analysed using anti-human LCN2 antibodies under non-reducing conditions to visualize both monomeric and dimeric species. Middle panel: all of the LCN2 species were immunoreactive with human specific LCN2 antibodies. Reducing conditions. Bottom panel: as a loading control, each mutant was also detected by Coomasie staining (100 ng per lane); reducing conditions. (**b**) Comparison of native, K3 and K3Cys mutants. Native and K3 formed dimers, but K3Cys produced only monomers *in vitro* (left panel) and *in vivo* (right panel). K3 and K3Cys were excreted to a much greater extent than native LCN2. Urine was collected for 3 h after i.p. innoculation (100 μg in 100 μl PBS, right panel). Non-reducing conditions. Anti-human LCN2 antibodies. (**c**) Urinary excretion of Alexa568-labelled native, K3 and K3Cys proteins (100 μg protein per mouse with equal fluorescent intensity). The image shows urine collected from 0–20 min, and from 20 to 180 min. (**d**) Urine was collected over 6 h and LCN2 was quantified by immunoblot. Note that the excretion of K3Cys exceeded native LCN2 by nearly 10-fold (72±19%; *n*=68 versus 4.9±3%; *n*=6; *P*=0.0001). Mean±s.d. Statistical analysis was performed by Student's *t*-test.

To identify specific LCN2 mutants which could evaded renal reabsorption, we performed functional screening *in vivo* by inoculating mutant LCN2:Ent:^55^Fe and subsequently we quantified 3 h urinary excretion using immunodetection and iron counting. As shown in Fig. 3a and Supplementary Fig. 2a,b, we found 13 LCN2 mutants that could load with Ent:^55^Fe and then could traffic through the kidney into the urine, particularly K3 (19% of input). Moreover, a number of the mutants, including K3 demonstrated little retention in kidney or liver (<2%; <1%) (Supplementary Fig. 2c,d). In contrast, negligible amounts of protein and iron (0.06%) were excreted into the urine by native LCN2, despite adequate loading with Ent:^55^Fe (Supplementary Fig. 2b). Hence, K3 was chosen for optimization.

Human LCN2 can form homodimers or heterodimers (e.g., LCN2-MMP-9 (ref. 39)) which increase molecular weight and inevitably limits clearance through the glomerulus^35^. To optimize K3, we mutated Cys87 to serine to generate K3-C87S (‘K3Cys') protein^30^. Whereas native and K3 produced both monomeric (∼20 KDa) and the dimeric (∼40 KDa) species (Fig. 3b), K3Cys produced only a ∼20 KDa monomer, both *in vitro* and *in vivo*. When we introduced these forms into mice, we found that K3Cys demonstrated the best urinary excretion (Fig. 3b,c). For example, 72±19% (*n*=68) of immunoreactive K3Cys, but only 4.9±3% (*n*=6) of native LCN2 appeared in the urine over 6 h after inoculation (*P*=0.0001, Student's *t*-test; Fig. 3d). Morever, unlike native LCN2, K3Cys was not retained by the kidney but rather was excreted in the urine (Supplementary Fig. 2f).

### Trafficking of K3 and K3Cys mutants

**Figure 4:**
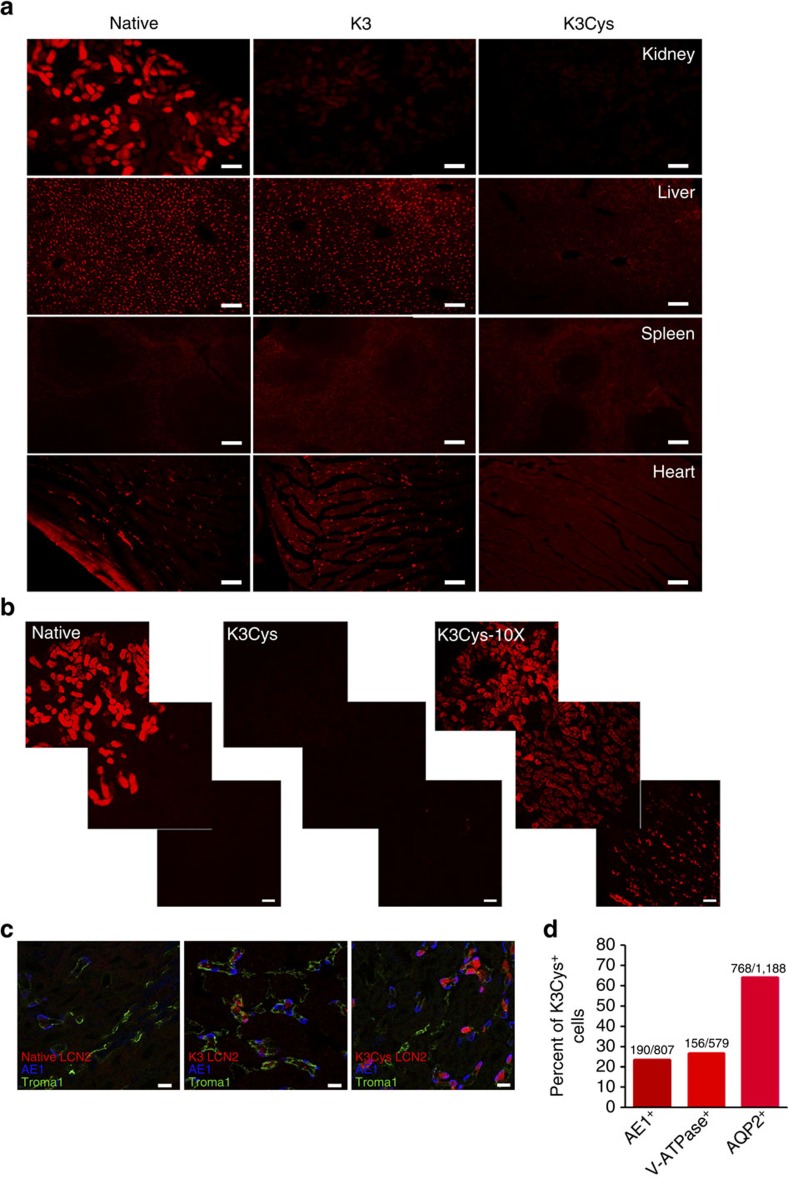
Trafficking of K3 and K3Cys mutants. Alexa 568-labelled native, K3 and K3Cys were introduced into mice (80 μg in 100 μl PBS; i.p.) and the retention of the labelled proteins was examined after 1 h. (**a**) Native Alexa 568-LCN2 was detected in kidney (proximal tubules), liver (Kupffer cells), spleen and heart, whereas K3 was nearly absent from the kidney but present in the other organs. K3Cys in contrast, was poorly visualized throughout the body. This is likely because K3Cys is rapidly and efficiently excreted. (**b**) Native Alexa 568-LCN2 was captured by the proximal tubule (left panel) whereas K3Cys was not visualized (middle panel). When the camera exposure time was increased (10 × ), K3Cys was found in the proximal tubule and in scattered cells in the medulla (right panel). (**c**) Analysis with AE1, V-ATPase and AQP2 immunocytochemistry demonstrated capture of K3Cys by principal and intercalated cells. There was limited capture of K3, and no evidence of native Alexa 568-LCN2 capture. (**d**) Speciation of K3Cys^+^ cells in kidney at the cortico-medullary junction. Scale bars **a**,**b**=100 μm; bars **c**=5 μm.

To remove iron through urinary excretion, K3Cys must be trafficked to the kidney for clearance without significant retention by cells of the kidney or of other organs. Therefore, we examined the trafficking of K3Cys and native LCN2 *in vivo* using labelled proteins. Consistent with the limited urinary clearance of unlabelled native LCN2, Alexa568- or fluorescein- labelled^26^ native LCN2 accumulated in the proximal tubule, rather than in the urine (Fig. 4a,b). In contrast, Alexa568-labelled K3 and K3Cys demonstrated almost no capture by kidney tubule cells. Over-exposure (10 × ) of the photomicrographs revealed only trace amounts of K3Cys in proximal tubule and in scattered medullary cells (Fig. 4b,c), including Aqp2^+^ Principal Cells (64.7% of Alexa568-LCN2 labelled cells) and AE1^+^ or ATPase^+^ intercalated cells (23.5% and 27.3%, respectively; Fig. 4d). Labelling with LCN2 was most intense in α-intercalated cells (native<K3<K3Cys), consistent with trafficking of K3 and K3Cys past the proximal tubule. In contrast, K3 and native LCN2 were captured to a similar extent by liver Kupffer cells, T-cell zones of the spleen and peri-myocytes, suggesting megalin-independent capture.

**Figure 5:**
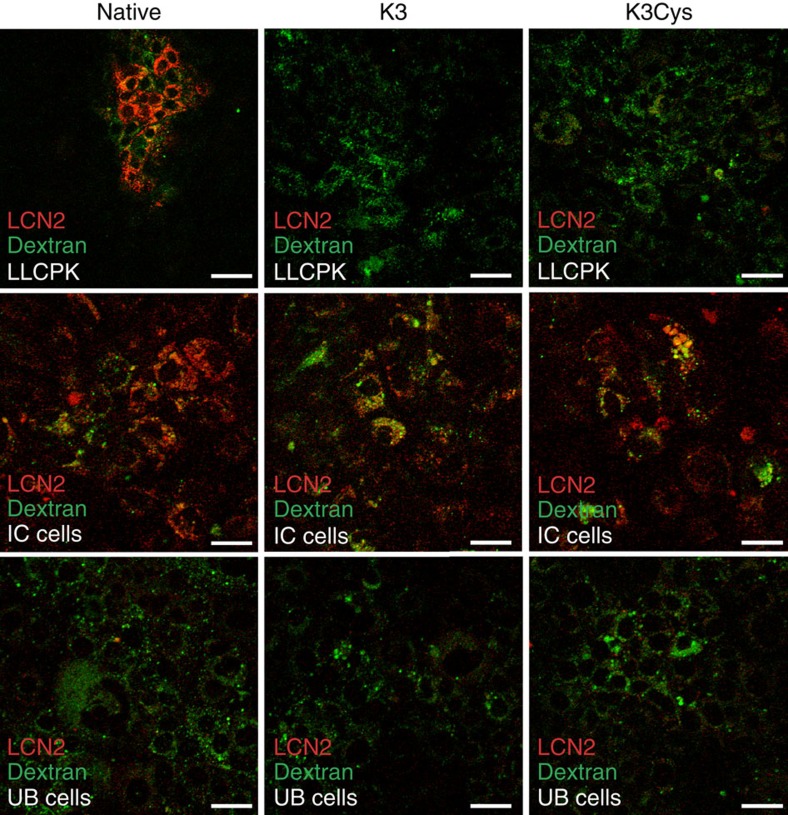
Capture of LCN2 by LLCPK, IC and UB cells. LLCPK (a proximal tubule cell line) captured Alexa568-native but not Alexa568-K3 or Alexa568-K3Cys proteins; IC (an intercalated cell line) captured Alexa568-native, Alexa568-K3 and Alexa568-K3Cys proteins; UB (an embryonic Ureteric Bud cell line) did not capture any of these proteins. Uptake of FlTC-dextran demonstrated active endocytosis. Scale bars, 50 μm.

To evaluate cellular trafficking in megalin^+^ and megalin^−^ cells, we utilized megalin^+^ LLCPK proximal tubule cells^40^, megalin^−^ intercalated cells^41^ and megalin^−^embryonic collecting duct UB cells^42^. All of these cells captured the fluid phase marker, fluorescent dextran, demonstrating that they were endocytically active, but LLCPK could capture only native LCN2, but not K3 or K3Cys. In contrast, intercalated cells captured all forms of LCN2, whereas UB cells failed to capture any form (Fig. 5). Hence, megalin expression (LLCPK cells) selectively captured wild type, but not K3 or K3Cys LCN2.

### K3Cys binds Ent:iron with high affinity

**Figure 6:**
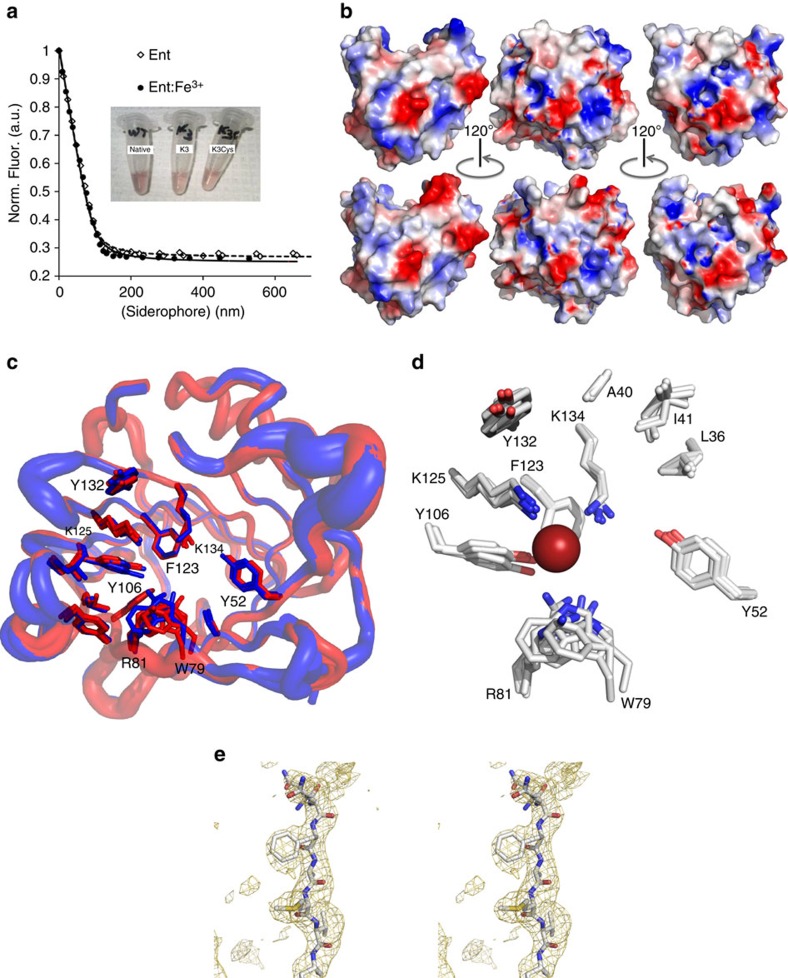
K3 and K3Cys bound Ent and Ent:Fe^3+^. (**a**) Fluorescence quenching binding assays at 340 nm demonstrated that both native and K3Cys had comparably high affinity for Ent (---) and for Ent:Fe^3+^ (**−**). The three tubes contain different species of LCN2:Ent:Fe^3+^. (**b**,**c**) Crystallography demonstrated that the calyx for Ent/Ent:Fe^3+^ binding was structurally conserved between K3 and native proteins. (**b**) Views of the molecular surfaces of native (top) and K3 (bottom) at different angles, coloured by electrostatic potential. These views show the structural effects of resurfacing. (**c**) Shows superposition of native LCN2 (red) with B-factor tubes for the backbone plus the two views of the K3 (blue) structure, highlighting the side-chains of residues around the binding site. (**d**) The big red sphere is the position of the iron atom from a bound Ent. All of these side-chains are highly structurally conserved. (**e**) Representative omit map shows the electron density around Phe 118, calculated with Fobs-Fcalc Fourier coefficients, contoured at 2sigma.

To efficiently excrete iron, LCN2 must not only target urine, but it must bind both Ent and Ent:iron, and retain the bound complexes over a broad pH range. Native, K3 and K3Cys LCN2 had similar Ent:Fe^3+^ binding activities, and remarkably retained Fe^3+^ with reddish colour typical of *tris*-catechol:Fe^3+^, even after 3 days of repetitive washing in acidified buffers over a pH range typical of urine (pH 4.5–7.5), (Supplementary Fig. 2a,e). Fluorescence quenching binding assays^30^,^43^ demonstrated that K3Cys bound Ent and Ent:Fe^3+^ with affinities comparable to native LCN2. Fits of stoichiometric binding curves estimated the dissociation constants to be *K*_D,Ent_=3.2±0.3 nM and *K*_D,Ent:Fe_=3.6±0.4 compared to *K*_D,Native_=0.4±0.1 (refs 30, 31, 32, 33) (Table 1; Fig. 6a). Therefore, K3Cys was capable of forming stable complexes with Ent:iron.

### The structural basis of the interaction of K3 and Ent

To define the structural basis for iron trafficking by K3, we solved its crystal structure (Table 2). Views of the molecular surfaces of LCN2 (top) and K3 (bottom) coloured according to electrostatic potentials, showed the large effect on the chemical character of LCN2 by the resurfacing mutations (Fig. 6b). Nonetheless, superposition of native and K3 structures demonstrated a high degree of overall structural conservation, including complete conservation of the ligand-binding site (Fig. 6c,d). In other words, neither the fold nor the ligand-binding site of LCN2 were affected by the mutation of surface residues, allowing K3 and K3Cys to efficiently traffic Ent:iron. A representative omit map showing electron density around Phe 118 is shown (Fig. 6e).

### Native and K3Cys quenched Ent:iron toxicity

We previously found that catechol could reduce Fe^3+^ to Fe^2+^, and the latter could catalyse the Fenton reaction. The addition of native LCN2, however, quenched the conversion^27^. Ent might also have redox activity, despite its affinity for iron (*K*_D_=10^−49^). We detected phenanthroline reactive Fe^2+^ after incubating Fe^3+^ with either Ent or mono-catechol (Supplementary Fig. 3). The reduced iron subsequently activated the conversion of 3′-(*p*-hydroxyphenyl) fluorescein (HPF) to fluorescein in the presence of H_2_O_2_. The addition of native or K3, however completely blocked the Ent- or catechol-mediated conversion of Fe^+3^ to Fe^+2^ (*P*<0.05±LCN2 with either Ent or catechol:iron, Student's *t*-test; *n*=3 each) and the subsequent oxidation of HPF to fluorescein (*P*<10^−7^, Student's *t*-test, *n*=3, across all points). In contrast, neither native or K3 activated HPF in the absence of Ent or mono-catechol. Hence, both native and mutant species of Scn could efficiently quench the chemical toxicity of Ent:Iron and catechol:Iron.

### K3Cys:Ent chelates transferrin-bound iron

Our data suggested that urinary excretion of iron could be enhanced by combining the high affinity of Ent for iron with its targeting to the urinary space by K3 proteins. However, iron is sequestered in different chemical pools, including circulating Tf and the heterogeneous NTBI pools (for example, citrated iron^44^). To determine whether Scn could efficiently chelate iron from Tf (*K*_D_=10^−23^ M)^45^, we incubated ^55^Fe loaded Tf in 1:1 stoichiometry with LCN2, and demonstrated iron transfer within 10 min of co-incubation. Iron transfer was specific because the complex had to include Ent (Supplementary Fig. 4a–c). We examined the same conditions *in vivo*. Inoculation of 12.5 nmoles of Tf-^55^Fe resulted in urinary recovery of only 0.02±0.02%, but the inoculation of 12.5 nmoles of Tf-^55^Fe and 25 nmoles K3Cys:Ent resulted in 18±6% urinary excretion of iron (*P*=0.002, Student's *t*-test; *n*=5). Similarly, inoculation of iron citrate resulted in the urinary excretion of only 1.9±2%, but the subsequent inoculation of 25 nmoles K3Cys:Ent resulted in the excretion of 24±8% of iron (*P*=0.0019, Student's *t*-test; *n*=5; Supplementary Fig. 4d). These data were particularly striking because neither Tf-^55^Fe nor Citrate-^55^Fe alone could label the kidney^27^. Consequently, K3Cys:Ent can capture and transfer iron into the urine from either exogenous Tf or Citrate.

### Iron overloaded mouse models

**Figure 7:**
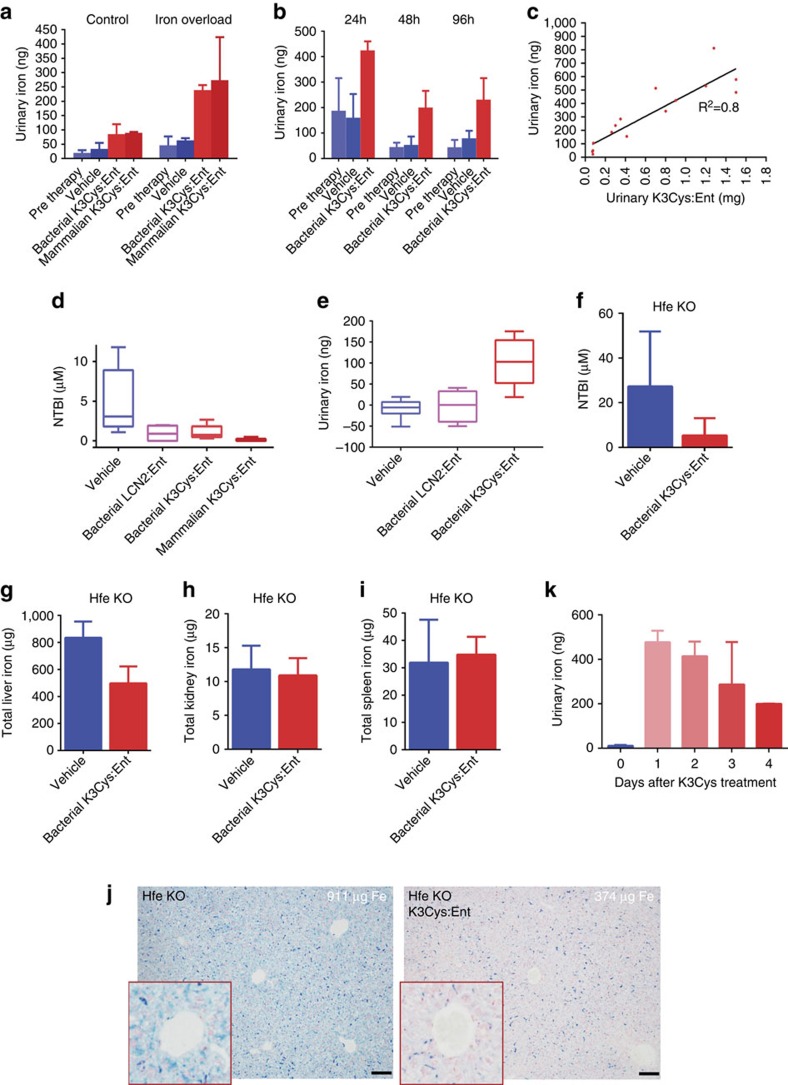
K3Cys:Ent transferred iron to the urine in murine models of experimental iron overload. (**a**–**c**) Urine was collected for 3 h after i.p. treatment of CFW mice with K3Cys:Ent (0.5 mg in 0.5 ml PBS per mouse) or an equal volume of saline (‘Vehicle'). (**a**) Total urinary iron was significantly increased by bacterially or mammalian expressed K3Cys whereas saline was not effective in increasing urine iron. (Control (*n*=26 assays): Pre therapy versus Saline *P*=0.48; Saline versus Bacterial K3Cys *P*=0.03; Saline versus Mammalian K3Cys *P*=0.009; Mammalian K3Cys versus Bacterial K3Cys *P*=0.4. Iron Overload (with 0.5 mg g^−1^ iron-dextran; *n*=18 assays): Pre therapy versus Saline *P*=0.12; Saline versus Bacterial K3Cys *P*=0.002; Saline versus Mammalian K3Cys *P*=0.05; Mammalian K3Cys versus Bacterial K3Cys *P*=0.77.) K3Cys:Ent exported iron to the urine at different time points. (**b**) Time course of urinary iron excretion. Saline was ineffective (*P*=NS), whereas K3Cys resulted in iron export at every time point (at 24 h: *n*=18 assays; Pre therapy versus Bacterial K3Cys *P*=0.0005, Saline versus Bacterial K3Cys *P*=0.0005; at 48 h: *n*=15 assays; Pre therapy versus Bacterial K3Cys, *P*=0.051; Saline versus Bacterial K3Cys *P*=0.041; at 96 h: *n*=20 assays; Pre therapy versus Bacterial K3Cys *P*=0.0067, Saline versus Bacterial K3Cys *P*=0.03). (**c**) Increasing dose of K3Cys protein increased the yield of urinary K3Cys and urinary iron. (**d**,**e**) Native LCN2 and K3Cys (0.5 mg) reduced serum NTBI by 4-fold after the transfusion of stored blood (*n*=24 assays: Saline versus native LCN2 *P*=0.046; Saline versus Bacterial K3Cys *P*= 0.050; Saline versus Mammalian K3Cys *P*=0.021) but only K3Cys exported iron to the urine, (**e**) The difference between treatment and pre-treatment urinary iron is shown (*n*=17 assays: Saline versus native LCN2 *P*=0.78; Saline versus Mammalian K3Cys *P*= 0.001; native LCN2 versus Mammalian K3Cys *P*= 0.006. Values represent median, IQR, minimum and maximum values. (**f**–**k**) *Hfe* mouse model. After 4 days of treatment with K3Cys:Ent (15 mg daily dose), the level of NTBI was reduced (*n*=4; *P*=0.030) and liver iron was reduced 40% (*n*=4; *P*=0.02), while spleen and kidney were unaffected (*n*=4 each; *P*=0.71–0.75). Reduced liver iron could be detected by comparing the most and least iron-saturated livers. K3Cys directed iron to the urine in *Hfe*^*−/−*^ mice, especially on days 1 and 2 of treatment. Mean±s.d. Statistical analysis was performed by Student's *t*-test. Scale bars **j**=100 μm.

Given that K3Cys:Ent complexes successfully excreted iron from exogenous donors, we examined whether K3Cys:Ent could excrete endogenous iron. We generated an iron overloaded mouse model with a single dose of iron-dextran (0.5 mg g^−1^), which was sufficient to elevate serum NTBI (11.6±4 μM; *n*=3, at 96 h), and then subsequently treated these mice with a single dose of either K3Cys:Ent (0.5 mg) or saline. We found that both bacterial and mammalian expressed K3Cys elevated urinary iron compared with saline treatment (*P*=0.03 and *P*=0.009, Student's *t*-test, respectively; *n*=4; Fig. 7a). K3Cys was effective on the day of iron load, as well as 4 days later (Fig. 7b). The stoichiometry of the urinary complexes (K3Cys:Fe) was consistent over 4 days of sampling (2.4±1, *n*=14; corrected for loading efficiency=1.5–1.7; see Methods). To determine whether the export of iron by K3Cys reflected endogenous iron content, we treated native mice and found that iron was still exported into the urine (Fig. 7a) but K3Cys was half-saturated (LCN2:Fe=7±1; corrected 5.0–4.4) compared to iron supplemented mice (*P*=0.00027, Student's *t*-test; *n*=8). These data demonstrated that the availability of endogenous iron determined iron capture.

Next we determined whether the amount of urinary iron was proportional to K3Cys therapy. In iron loaded mice, we recovered an 11-fold range of urine iron for an 18-fold range of urine LCN2 (*R*^2^=0.8; *n*=14; Fig. 7c) with constant protein–iron stoichiometry (3.2±1.6; corrected 2.3–2.0). In contrast, equal volumes of saline had little effect on iron excretion compared with pre-treatment urine samples (24–96 h, *P*=0.061–*P*=0.73, NS, Student's *t*-test; Fig. 7a,b).

The leading cause of secondary iron overload is chronic blood transfusion. Stored blood in particular can elevate serum NTBI and tissue iron. To determine whether LCN2:Ent could clear post-transfusion iron, we used an autologous mouse transfusion model^46^. LCN2:Ent was introduced 4 h after transfusion when NTBI was known to peak, and blood and urine were sampled after a 3 h interval. We found that both bacterially and mammalian expressed K3Cys (0.5 mg) effectively delivered iron into the urine (*P*=0.0015, Student's *t*-test; *n*=17), but saline (*P*=0.33, Student's *t*-test) and wild-type LCN2 (*P*=0.33, Student's *t*-test) were not effective. LCN2 species also reduced NTBI (saline versus wild type or K3Cys, *P*=0.047 and *P*=0.032, respectively, Student's *t*-test; *n*=24; Fig. 7d,e).

The leading cause of hereditary haemochromatosis is the mutation of *Hfe*. We treated 12 week old *Hfe* null mice (which are iron overloaded by 4 weeks of age) with four doses of K3Cys over 4 days *n*=3, and found decreased serum NTBI (*P*=0.030, Student's *t*-test; *n*=7; Fig. 7f), and a 40% reduction in liver iron (*P*=0.02, Student's *t*-test; *n*=7; Fig. 7g), without changes in kidney or spleen iron (*P*=0.71, Student's *t*-test, 0.75 respectively, Fig. 7h,i). The reduction in hepatic iron load was visible by Perl's stain when comparing the most iron saturated, untreated liver with the least iron-saturated liver after treatment (Fig. 7j). Removal of iron was co-incident with a 50-fold increase in urine iron, particularly on the first and second days of treatment (*P*=0.0001, Student's *t*-test; *n*=7; Fig. 7k).

## Discussion

DFO produced by *Streptomyces pilosus* (*K*_D_=10^−34^) was the first bacterial siderophore used in humans to treat iron overload^34^. Nonetheless DFO (and synthetic iron chelators Deferiprone and Deferasirox) can incur severe toxicity, or stimulate infection by delivering iron to pathogenic bacteria and fungi. Ent produced by *E. coli* (*K*_D_=10^−49^) is also a siderophore, but efforts over the past half century to use Ent therapy have failed because of its instability and toxicity, inappropriate targeting to intestine and liver, and donation of iron to bacteria^47^,^48^.

Here we evaluated a novel strategy for iron chelation utilizing a carrier protein. We first considered several different iron binding proteins, but none were suitable for the definitive excretion of iron. Tf for example, chelates iron with high affinity (*K*_D_=10^−23^ M, pH=7.4), but it also delivers iron to many types of cells by binding its receptor, TfR1. Tf would also release its bound iron in the collecting ducts where urine pH falls below 6.0, rather than deliver iron to the urine in an intact complex. Lactoferrin (*K*_D_=10^−20^ M) was also a candidate—it has a similar functional structure as Tf (ref. 49)—but like Tf, its iron is recaptured through receptors found in brain, liver, bone and lymphocytes^50^, and its size (∼80 KDa) precludes renal excretion. Ferritin is the major iron binding protein in the cytoplasm, and it perhaps transports iron in the serum in iron overloaded conditions. Although this system is attractive, since one complex can bind up to 4,500 irons, ferritin can nonetheless be recaptured via Timd2 or Scara5 receptors, and it cannot be cleared by kidney filtration due to the polymerization of its monomers into very large molecular weight complexes^51^.

Similar to Tf, Lactoferrin (Lf) and ferritin, LCN2 can associate with iron at high affinity, but it does so through catecholate cofactors, such as Ent or the ‘monomeric' catechols. The catechol groups have high affinity for iron when three are present to fully coordinate (hexadentate) iron, which can occur by loading three monomeric catechols into the LCN2 calyx^27^, or through scaffolding on an intramolecular backbone (Ent). While monomeric catechols dissociate from LCN2 at low pH, Ent remains bound. As a result, LCN2:Ent is a superior, high affinity, low toxicity chelator appropriate for the urinary space (pH 6.5–4.5).

LCN2 is also a small protein (∼25 kDa) which can be introduced by subcutaneous inoculation, followed by its rapid filtration through the kidney's glomerulus. Because a direct interaction with megalin was shown by SPR^52^ and the deletion of megalin from the proximal tubule resulted in the appearance of urinary LCN2, we predicted that the surface positive residues of LCN2 (which are thought to interact with megalin) could be manipulated to permit iron excretion. Hence, while additional receptors must be present in proximal tubule and collecting ducts to account for residual uptake (in agreement with Thévenod^53^) they cannot compensate for the disruption of megalin–LCN2 interactions. In sum, biochemical, cellular and animal data, suggested the hypothesis that K3 and K3Cys bypassed the proximal tubule as a result of a failure to recognize megalin.

These data provided a strategy to remove excessive iron, because LCN2:Ent could be diverted into the urine. For each gram of K3Cys (50 μmoles) given to an iron overloaded patient, 50 μmoles (2.8 mg) of iron could be excreted (assuming 100% clearance of K3Cys and a molar ratio of 1:1:1 for LCN2:Ent:Iron). Consequently, this is an efficient way to remove excessive iron from a human patient because LCN2:Ent traffics and disposes approximately the same amount of iron as the daily iron loss or gain at steady state (1–2 mg per day). In addition, mutant LCN2:Ent complex might be useful not only as a therapy but also in the prophylaxis of blood transfusions. We suggest that our strategy may be further optimized with a stable form of Ent, known as TRENCAM^54^ which binds LCN2 (LCN2:TRENCAM:Fe) or by the production of low molecular weight protein scaffolds to limit the amount of protein required to guide the siderophore past megalin into the urine.

While K3Cys:Ent was able to capture iron directly from transferrin and from citrate and deliver it to the urine, we have not resolved the endogenous source of iron for clearance. Endogenous pools of iron integrate many different stimuli and can be modified not only by iron chelators but also by inflammatory and hormonal stimuli (for example, by upregulating hepcidin). Consequently, while LCN2 reduced NTBI, suggesting that it withdrew iron from the NTBI pool, signalling by LCN2 protein itself or by contaminants such as LPS (even when reduced by Triton-114 and Polylysine extraction), could modify the partitioning of iron and reduce NTBI independently of iron chelation. Hence the exact source of iron (serum iron, NTBI, Tf-bound-iron, cellular iron) delivered to the urine by LCN2:Ent needs to be resolved in future experiments. In addition, K3 may not remove iron from all cell types. For example, K3 is readily endocytosed by Kupffer cells (Fig. 4a), perhaps accounting for their iron load even after treatment with high doses of K3Cys LCN2:Ent (Fig. 7j). The idea that an iron chelator can deliver iron to subsets of cells is consistent with studies of other iron-carrying proteins^55^,^56^.

In summary, LCN2 protein is expressed in the plasma and urine when sepsis, ischaemia or nephrotoxins injure the kidney^57^. Our data demonstrates that megalin blocks the passage of plasma LCN2 into the urine, creating two independent pools of LCN2 (plasma and urine). Nonetheless, multiple mutations of surface residue resulted in megalin bypass, allowing the passage of the mutant iron scavenger into the urine. Fortunately, K3 maintains almost all structural and functional features of the native protein permitting iron transport. The monomer K3Cys together with its ligand Ent can chelate endogenous iron and deliver the bound iron to the urine, confirming this proposed novel iron chelation and urinary excretion strategy.

## Methods

### Production of LCN2

Human LCN2 ORF without N-terminal signal peptide coding sequence was PCR-amplified from *LCN2* cDNA clone (Genbank accession number: NM_005564, Open Biosystems) and then subsequently cloned into the BamHI and EcoRI sites of pGEX-4T-3 (GE Healthcare) to generate a pGEX-4T-3-LCN2-GST. The latter was used as a template for site-mutagenesis of Scn using Quick-Change Site-Directed Lightning Multi-Mutagenesis kit (Stratagene).

Plasmids containing native or mutant *LCN2* were electroporated into BL21 *E. coli* (GE Healthcare). Protein was then produced by IPTG (0.2 mM, 5 h) induction and purified from the bacteria by a GST-based affinity purification system (Glutathione Sepharose) and gel filtration (Hi-Prep 16/60 Sephacryl S-100HR, GE Healthcare)^58^. To produce Apo-LCN2, iron sulfate (100 μM) was added to suppress the synthesis of Ent.

The bacterially expressed native and K3Cys proteins were stripped of LPS by combining Triton X-114 extraction and affinity removal. Briefly, Triton X-114 was added to native and K3Cys (20 mg ml^−1^) at 1% final concentration; the mixture was then shaken vigorously, incubated at 4 °C for 30 min with rotating and then 37 °C for 10 min followed by centrifugation (20,000*g* for 10 min at 25 °C). The upper aqueous phase containing LCN2 was collected and three additional extractions were performed. Residual LPS in the LCN2 solution was removed using Pierce High Capacity Endotoxin Removal Columns (Thermo Scientific). This protocol allowed us to recover >75% of the initial protein and consistently reduce LPS to ∼0.5 EU mg^−1^ protein (LAL Assay Test Kit; Thermo Scientific).

To produce LCN2 in mammalian cells, human LCN2 ORF was cloned to pcDNA3.1(+) to generate pcDNA3.1(+)-*LCN2* which was then transfected into Freestyle 293-F cells using 293fectin (Life Technologies). Stably transfected cell lines were selected using G418 (800 μg ml^−1^) and the culture medium was collected and concentrated for purification by using LPS-free column chromatography (Supplementary Fig. 5).

### Preparation of LCN2:Ent:Fe complex

The LCN2:Ent:Fe complex was prepared by mixing LCN2 and iron-saturated Ent (EMC Collections or Sigma) at a 1:1 ratio. Unbound Ent or Ent:Fe was removed by ultrafiltration in a 10 K microcon with four washes using a saline solution (NaCl 150 mM, KCl 4 mM, HCO_3_ 5 mM, pH 7.4). We measured a molar ratio of 0.72 total iron to LCN2 protein (by atomic absorption and immunoblot, respectively) in the saturated LCN2:Ent:Fe complex, which demonstrated that 72% of bacterially expressed protein had functional Ent binding activity. Alternatively, when the complex was prepared by mixing equimolar amounts of LCN2, Ent, and ^55^Fe (1:1:1), 63.2±6.2% of ^55^Fe was protein bound (*n*=17), further confirming high production efficiency, but also implying that a portion of protein (∼37%) was already occupied by Ent or partially degraded Ent. Based on these stoichiometric analyses, the amount of LCN2 to bind Ent was corrected to be a 63–72% maximal efficiency.

### Iron capture in iron overloaded mouse models

Male mice (CFW, 10 weeks) were injected once with iron-dextran (Sigma-Aldrich, 0.5 mg g^−1^ in 500 μl of 0.9% NaCl, *n*=3) after passage through a PD-10 column, and LCN2 was introduced i.p. 48 h later. Alternatively, mouse RBCs were collected and stored as previously described^46^ and used to iron load mice. Briefly, whole blood was collected from 20 to 50 C57BL/6 mice in CPDA-1 solution and leukoreduced using a Neonatal High-Efficiency Leukocyte Reduction Filter (Purecell Neo; Pall Corporation). The isolated RBCs (∼10 ml) were packed to ∼60% haematocrit and refrigerator stored for up to 14 days. RBC suspensions (400 μl at 17.0–17.5 g dl^−1^ haemoglobin; 2 equivalent human units, respectively) were transfused through the retro-orbital plexus of isoflurane-anesthetized mice (C57BL/6, 8 weeks). In a third model, 10-week old male *Hfe* homozygous knockout mice (Jackson, Strain 129S6-SvEvTac) were placed on 42 p.p.m. iron diet (Harlan) for 2 weeks and then treated with K3Cys:Ent (15 mg per day in divided doses) or Saline.

K3Cys and other forms of LCN2 were introduced, and urine was collected in 3 h intervals. LCN2 was measured by immunoblot (Bioporto) using bacterial protein for standards. The kidney and liver were dissected and solubilized in a solution with 0.2 mM NaOH and 10% SDS for counting or for non-haem iron measurements. Liver was also fixed in 4% PFA in PBS for 24 h followed by 70% EtOH for Perls staining. Endogenous urine iron was measured at the Trace Metals Core Facility at the Mailman School of Public Health of Columbia University using atomic absorption with a Perkin-Elmer Analyst 600 graphite furnace system. NTBI was measured according to the nitrilotriacetic acid chelation and ultrafiltration assay^46^. All animal experiments followed protocols approved by the IACUC at Columbia University.

### *In vivo* capture of native and K3

LCN2 was fluorescently labelled with Alexa568-Succinimidyl Ester (Molecular Probes) by following previous protocols^58^ and instructions from the manufacturer (https://tools.thermofisher.com/content/sfs/brochures/TR0031-Calc-FP-ratios.pdf) including clean up by gel filtration followed by microcon (10 K) washes. The molar ratios of Alexa dye to native, K3, K3Cys proteins were 0.98, 1.20 and 0.73. The proteins were introduced (80 μg for LCN2, 8-week old mice) and after 1 h, kidney, liver, heart and spleen were fixed in 4% PFA. Immunohistochemical staining of the kidney proximal tubules (Anti-Megalin, Santa Cruz; LTL, Vector Labs) collecting ducts (Anti-Tromal Ab, Developmental Studies Hybridoma; DSHB; Anti-AE1 Ab), Principal cells (Anti-AQP2), Intercalated cells (Anti-V-ATPase Ab) were visualized using Zeiss LSM 510 Meta Confocal Scanning Microscope. Immunoblots were assayed with anti-mouse LCN2 (R&D Systems) and anti-mouse Tf (Bethyl Laboratories, A90-129A).

### Crystallography

Protein was exchanged into 25 mM PIPES (pH=7) plus 150 mM NaCl, and concentrated to 10 mg ml^−1^. Diffraction-quality crystals were obtained by hanging-drop vapour diffusion, with a well solution of 1–1.2 M Na_2_HPO_4_/KH_2_PO_4_, pH=7.2. Drop size was 1 microliter of protein solution plus 1 microliter of well solution, prepared manually. Crystals were grown at room temperature. Diffraction data were collected at the Advanced Light Source (Berkeley, CA, USA) beamline 5.0.1 and integrated and scaled with Mosflm^59^. Initial phases were determined by molecular replacement using MolRep^60^ as implemented in the CCP4 software suite^61^, using all atoms in Chain A from 1L6M.pdb (ref. 30) as a search model. Iterative rounds of alternating positional refinement and model building, using the programs Refmac^62^ and COOT^63^, including placing ordered solvent molecules and phosphates, were followed by a final round of TLS refinement^64^. Residues or side-chains lacking corresponding electron density in Fourier syntheses with 2F_obs_–F_calc_ coefficients, contoured at 0.7σ, were removed or truncated to the Cβ atom. The quality of the final model was assessed using ProCheck^65^ and Molprobity^66^ (final overall score: 100th percentile). The final model has been deposited in the PDB^67^ with accession code 5JR8. Crystallographic statistics are reported in Supplementary Table 1.

### Fluorescence quenching titrations

Fluorescence measurements were performed as previously reported with minor modifications^43^. The Jobin Yvon Fluorolog fluorometer had 2 nm excitation slits, 5 nm emission slits, 280 nm excitation wavelength, and a 320–360 nm emission scan. The ligand solutions (6 μM siderophore, TBS with 5% (v/v) DMSO, pH 7.4) were prepared from 15 mM stock solutions. Absorbance measurements of the ligand solutions were performed before and after every titration to confirm the ligand concentration (ferric enterobactin *ɛ*_496=_5,600 M^−1^ cm^−1^; apo-enterobactin *ɛ*_316=_9,500 M^−1^ cm^−1^)^68^,^69^. Aliquots of the ligand solution were added to 3 ml of the protein solution (95 nM K3Cys, 9.5 μg ml^−1^ ubiquitin (Sigma), TBS, 4.75% DMSO, pH 7.4) in 1 × 1 cm cuvettes and mixed before measuring the fluorescence. The data from three titrations at 340 nm were corrected for dilution, normalized, and fit to a one-to-one binding model using the programme DYNAFIT^70^. The *K*_D_ values are reported with the calculated standard error in parentheses.

### Assays of chemical reactivity

Reduction of iron by Catechol or Ent was detected in a reaction mixture contained 0.24 M K phosphate buffer (pH 7.4), 30 mM sodium citrate, 15 μM FeCl_3_, 50 μM Catechols (in 50 mM potassium phosphate buffer, pH 6.5) and 5 mM o-phenanthroline or ferrozine. Iron reduction was monitored by absorbance (512 nm) 5 min after mixing using a Ultrospec 3300 pro UV/visible spectrophotometer at room temperature.

Hydroxyl radicals were detected by the conversion of HPF to fluorescein. The reaction mixture contained 0.20 M sodium phosphate buffer (pH 7.4), 30 mM EDTA, 10 μM FeCl3, 30 μM catechols or Ent, 2 mM H_2_O_2_, and 10 μM 3′-(p-hydroxyphenyl) fluorescein (HPF; Invitrogen) or 20 μM F1300 (Invitrogen). Hydroxyl radicals were detected by the conversion of HPF to fluorescein. After one hour at room temperature, the fluorescence of the reaction mixture was measured with a LS55 Luminescence Spectrometer, Ex 490 nm, Em 515 nm.

### Measurements of Inflammatory response

Cytokines/chemokines were quantified using the Cytometric Bead Array Mouse Flex Kit (BD Biosciences). Heparinized plasma obtained by cardiac puncture. Flow cytometric cytokine data (FACSCalibur flow cytometer; BD Biosciences) were analysed using FlowJo software (TreeStar).

### Statistical analysis

All values are presented as mean±s.d, unless otherwise specified. Box plots represent median and interquartile range (IQR), and whiskers represent minimum to maximum values. Student's *t*-test (two-tailed) was used for comparisons. Statistical analysis was performed using GraphPad Prism software (version 6).

### Data availability

Sequence data that support the findings of this study have been deposited in GenBank with accession code NM_005564. Structural data supporting the findings of this study have been deposited in PDB with accession code 5JR8. All the other data are available from the corresponding authors upon reasonable request.

## Additional information

**How to cite this article:** Barasch, J. *et al*. Disposal of iron by a mutant form of lipocalin 2. *Nat. Commun.* **7**, 12973 doi: 10.1038/ncomms12973 (2016).

## Supplementary Material

Supplementary Information Supplementary Figures 1-6 and Supplementary Table 1

## Figures and Tables

**Table 1:** Affinity (*K*_D_) for enterochelin.

	Apo-Ent (nM)	Ent:Fe^3+^ (nM)
**Native LCN2**	3.6 (2)	0.4 (1)
**K3Cys LCN2**	3.2 (3)	3.6 (4)

References 30,31,32,33.

**Table 2:** X-ray data collection and refinement statistics.

*Data collection*	
Space group	P4_3_2_1_2
*Cell dimensions*	
a=b, c (Å)	139.0, 64.9
Resolution (Å)	50.00–2.65 (2.78–2.65)*
*I*/σ*I*	18.3 (4.5)
Completeness (%)	100 (100)
Redundancy	8.8 (8.1)
*R*_merge_	7.6 (42.0)
*Refinement*	
Resolution (Å)	50.00–2.65 (2.78–2.65)
No. reflections	19,056 (2493)
*R*_work_/*R*_free_	18.1/20.9
*No. atoms*	
Protein	2,763
Ligand/ion	21
Water	55
*B-factors*	
Protein	53
Ligand/ion	65
Water	38
*R.m.s deviations*	
Bond lengths (Å)	0.017
Bond angles (°)	1.965

Data were collected from a single crystal.

^*^Highest resolution shell is shown in parentheses.
