# Supplementary material for: Disposal of iron by a mutant form of lipocalin 2
Source: Nat Commun. 2016 Oct 31;7:12973. doi: 10.1038/ncomms12973 (PMC5095531; doi:10.1038/ncomms12973)
Supplement: Supplementary Information — Supplementary Figures 1-6 and Supplementary Table 1 [file ncomms12973-s1.pdf]

Supplementary Figure 1

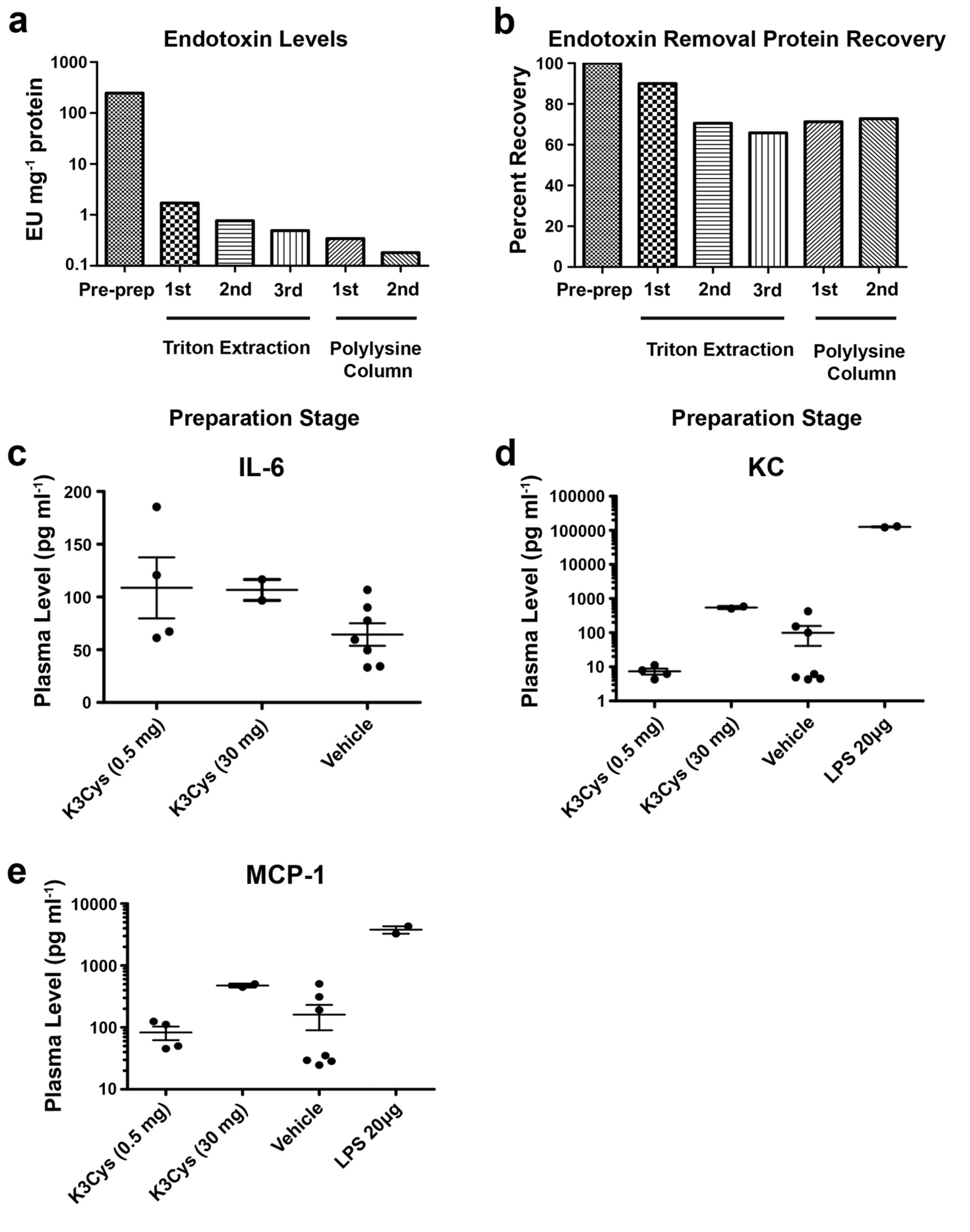

Supplementary Figure 1 | Protein clean-up. (a) LPS was removed from bacterially expressed native and K3Cys proteins by combining multiple Triton X-114 extractions and affinity chromatography (Pierce High Capacity Endotoxin Removal Columns). Recovery ~0.5 EU LPS mg^{-1} protein (LAL Assay Test Kit). **(b)** Recovery >75% protein. **(c-e)** Post injection induction of cytokine expression demonstrating protein and diluent had similar activities especially at low dose (n=3; saline vs LCN2, 30 mg, $p=0.092$ for KC; $p=0.12$ for IL-6 and $p=0.27$ for MCP1). Hepcidin levels were unchanged after a single dose of K3Cys (30 mg, data not shown). Abbreviations: EU, endotoxin unit. Mean±S.D. Statistical analysis was performed by Student's t-test.

Supplementary Figure 2

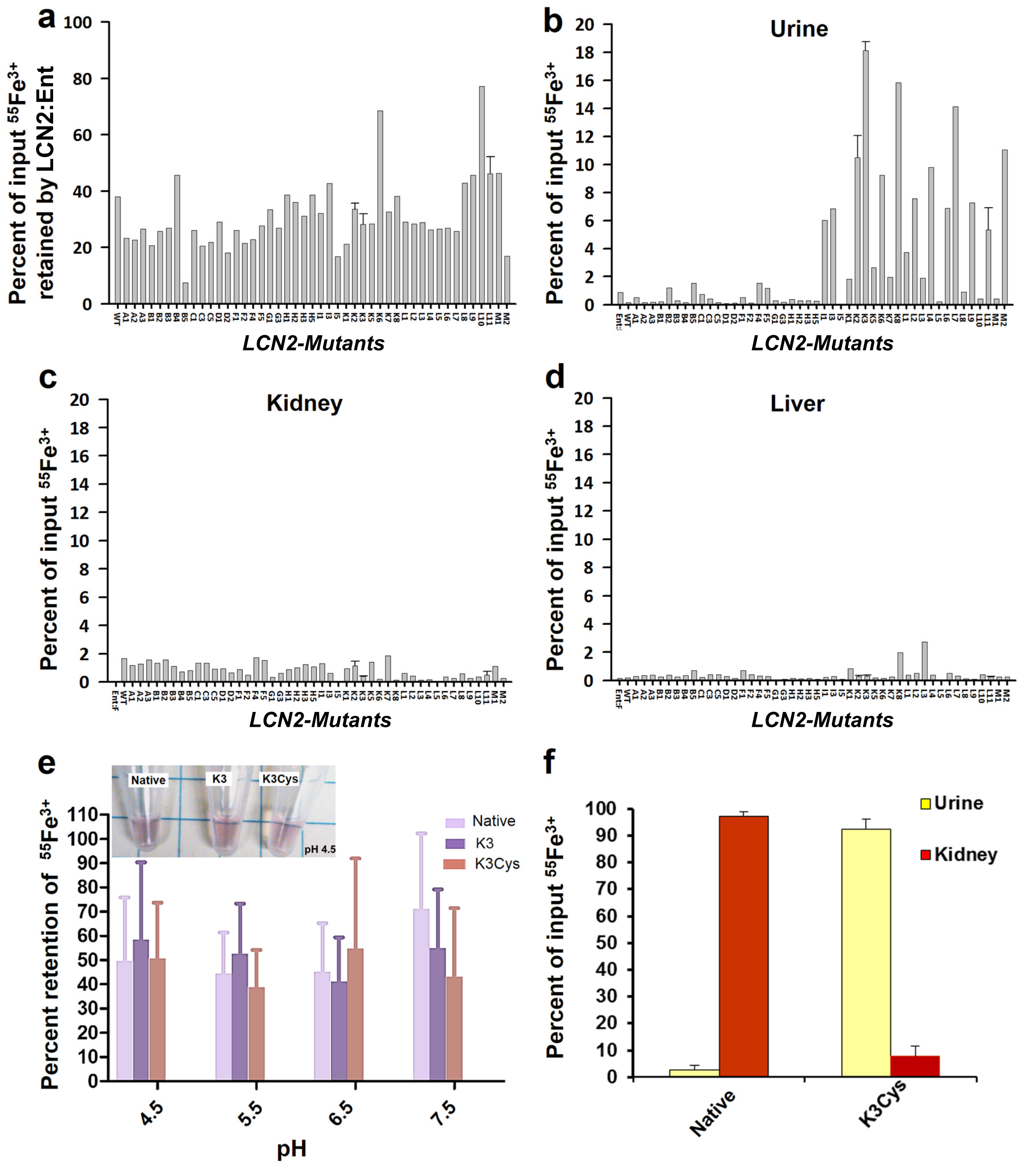

Supplementary Figure 2 | Screening LCN2 mutants for iron transport. (a) Mutant LCN2 proteins were screened by retention assays to identify whether the mutants could bind Ent and $^{55}Fe^{3+}$ (n=3). **(b-d)** The complexes (0.1 mg in 0.4 ml PBS) were then inoculated *i.p.*, and urine, kidney, and liver were collected 3 hours later. **(e)** Retention assays were performed by incubating equal moles of native, K3 or K3Cys and Ent and ^{55}Fe (1:1:1) at pH7.4 for 30 min, followed by quadruple washing with PBS buffers (pH4.5, 5.5, 6.5 or 7.5) each day for 3 days. There were no significant differences in Ent:Fe retention at different pH buffers or different LCN2 species. Note retention of the pink color (Ent:Fe) despite washing the protein in acidified buffer (n=8). **(f)** Kidney and urine were compared after inoculation with native or K3Cys:Ent:Fe3+ mutants. Native LCN2 targeted the kidney but could not deliver iron to the urine, while K3Cys did the reverse (Native n=2, K3Cys n=8; Native vs K3Cys kidney $p<10^{-7}$; Native vs K3Cys urine $p<10^{-7}$). Mean±S.D. Statistical analysis was performed by Student's t-test.

Supplementary Figure 3

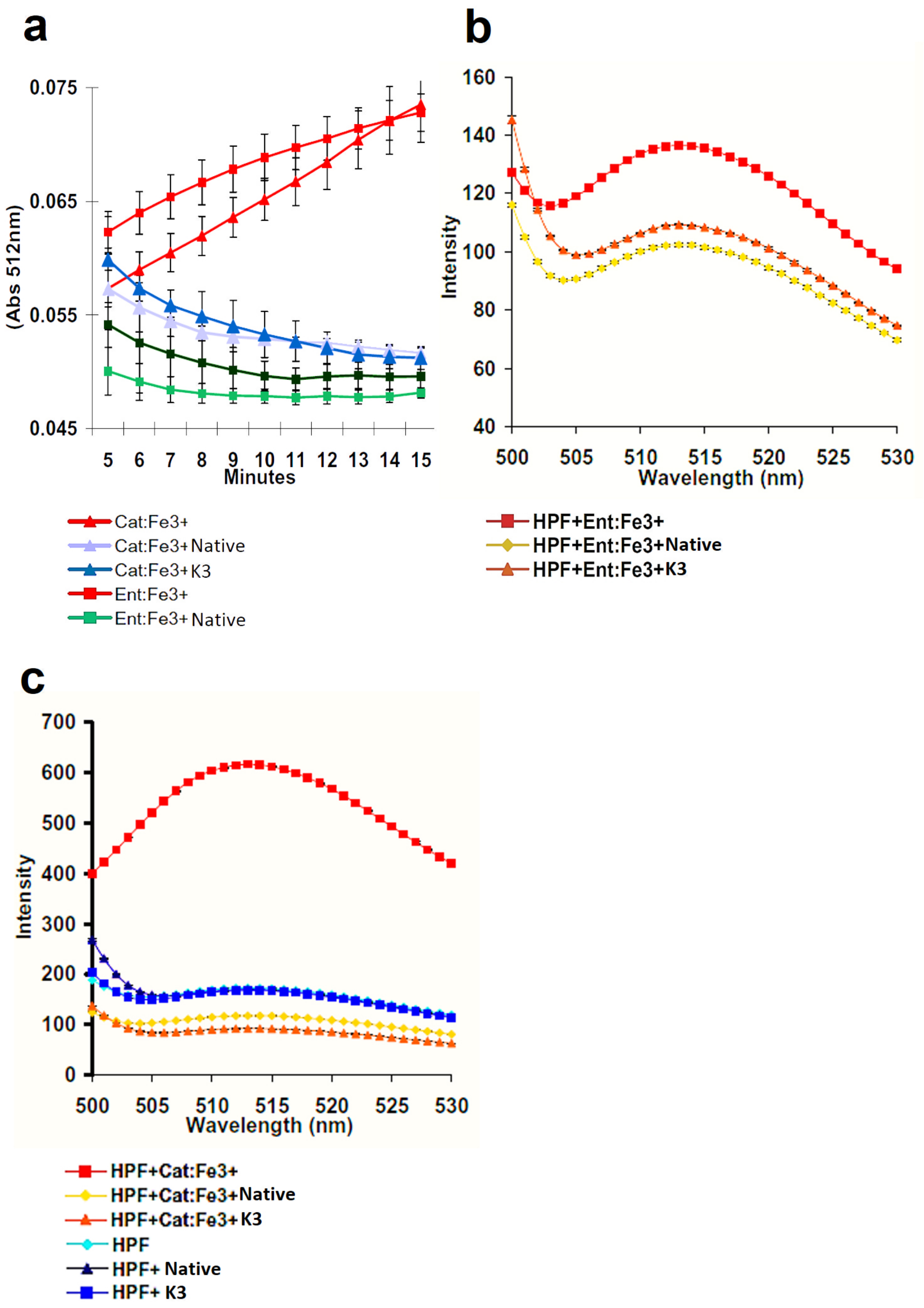

Supplementary Figure 3 | K3Cys limited iron reactivity. (a) Catechol and Ent converted Fe^{3+} to Fe^{2+}, but the addition of native or K3 limited the reduction of iron (lower curves K3Cys or native LCN2: $p \leq 0.02$, n=3, at time points >10 min) **(b)** Ent:Fe^{3+} activated HPF, but the addition of wild type or K3 quenched the activity (lower curves K3Cys or native LCN2: $p < 10^{-20}$, n=3, across all points). **(c)** Catechol:Fe^{3+} activated HPF, but the addition of native LCN2 or K3 limited HPF activation (lower curves K3Cys or native LCN2: $p < 10^{-7}$, n=3, across all points). Abbreviations: cat, Catechol. Mean±S.D. Statistical analysis was performed by Student's t-test.

Supplementary Figure 4

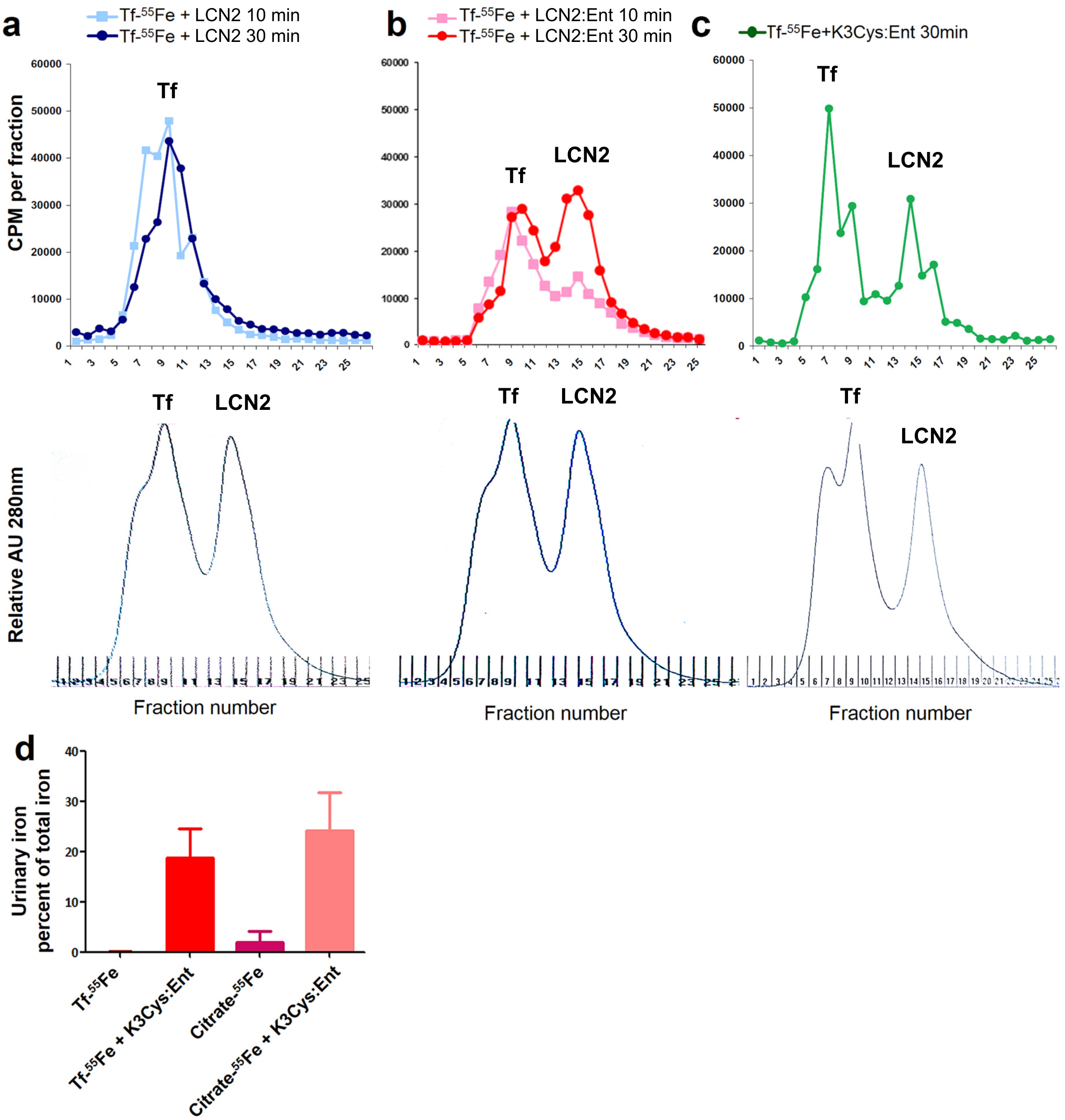

Supplementary Figure 4 | K3Cys:Ent chelates iron donated by Tf or by citrate. Tf:$^{55}Fe^{3+}$ was mixed with **(a)** LCN2, **(b)** LCN2:Ent or **(c)** K3Cys:Ent for 10 or 30 min. The complexes were then subjected to gel-filtration (Superdex-75) and fractions counted for $^{55}Fe^{3+}$ and protein concentration (280nm). Peaks for Tf and LCN2 are identified. **(d)** The inoculation of K3Cys:Ent (25 nmoles in 100 µl PBS; *i.p.*) redirected iron from Tf:$^{55}Fe^{3+}$ ($p=0.002$; $n=5$) or from citrate:$^{55}Fe^{3+}$ donors to the urine ($p=0.0019$; $n=5$). Mean±S.D. Statistical analysis was performed by Student's t-test.

Supplementary Figure 5

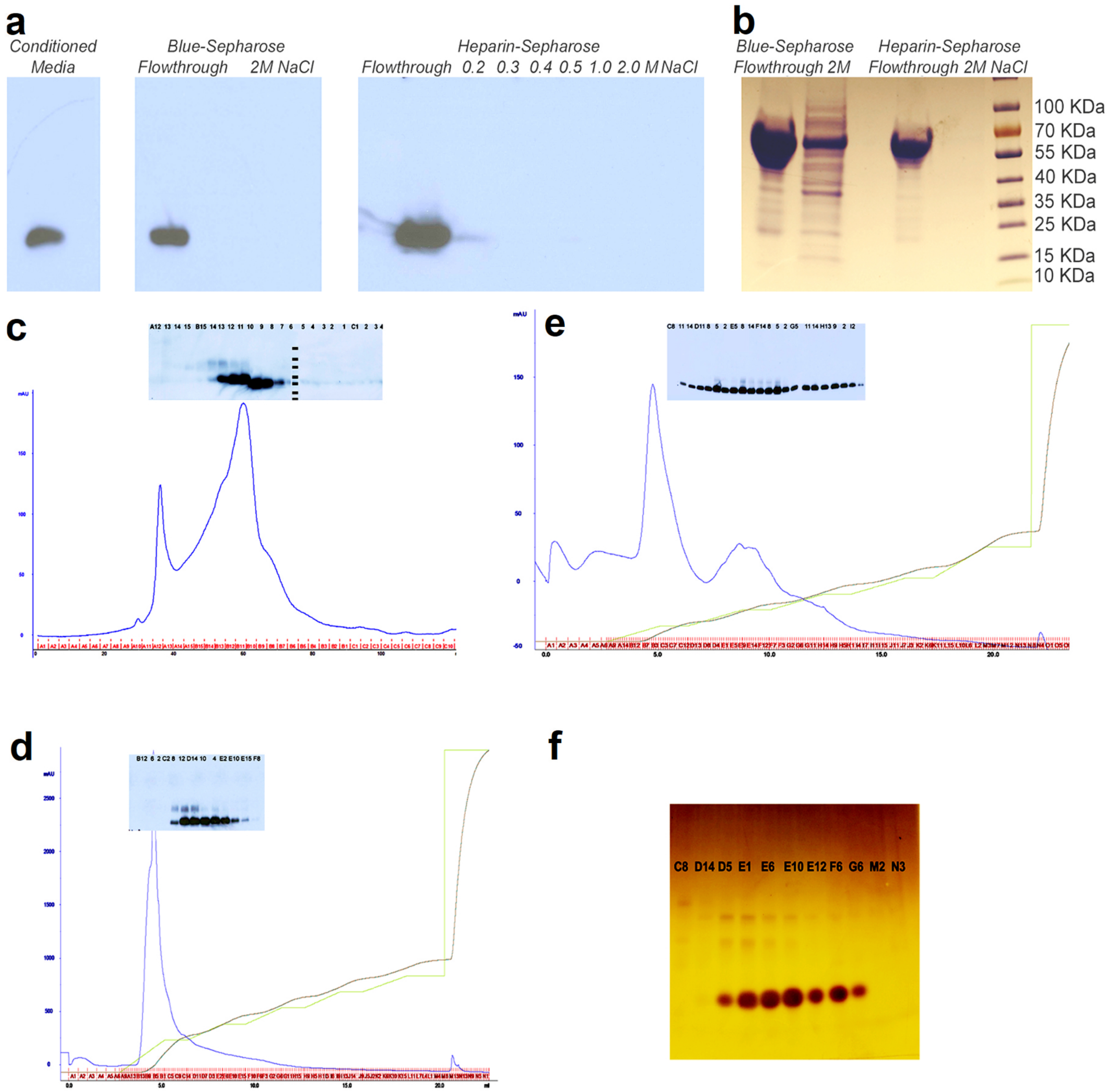

Supplementary Figure 5 | Purification of K3Cys protein expressed in 293 Free-style cells. Conditioned media was fractionated by **(a-b)** Blue Sepharose, Heparin Sepharose, **(c)** Gel Filtration followed by **(d-e)**. repetitive MiniQ chromatography. **(f)** Silver stained gels demonstrating the purification. K3Cys was followed through the purification by immunoblotting using anti-human LCN2 antibodies.

Supplementary Figure 6

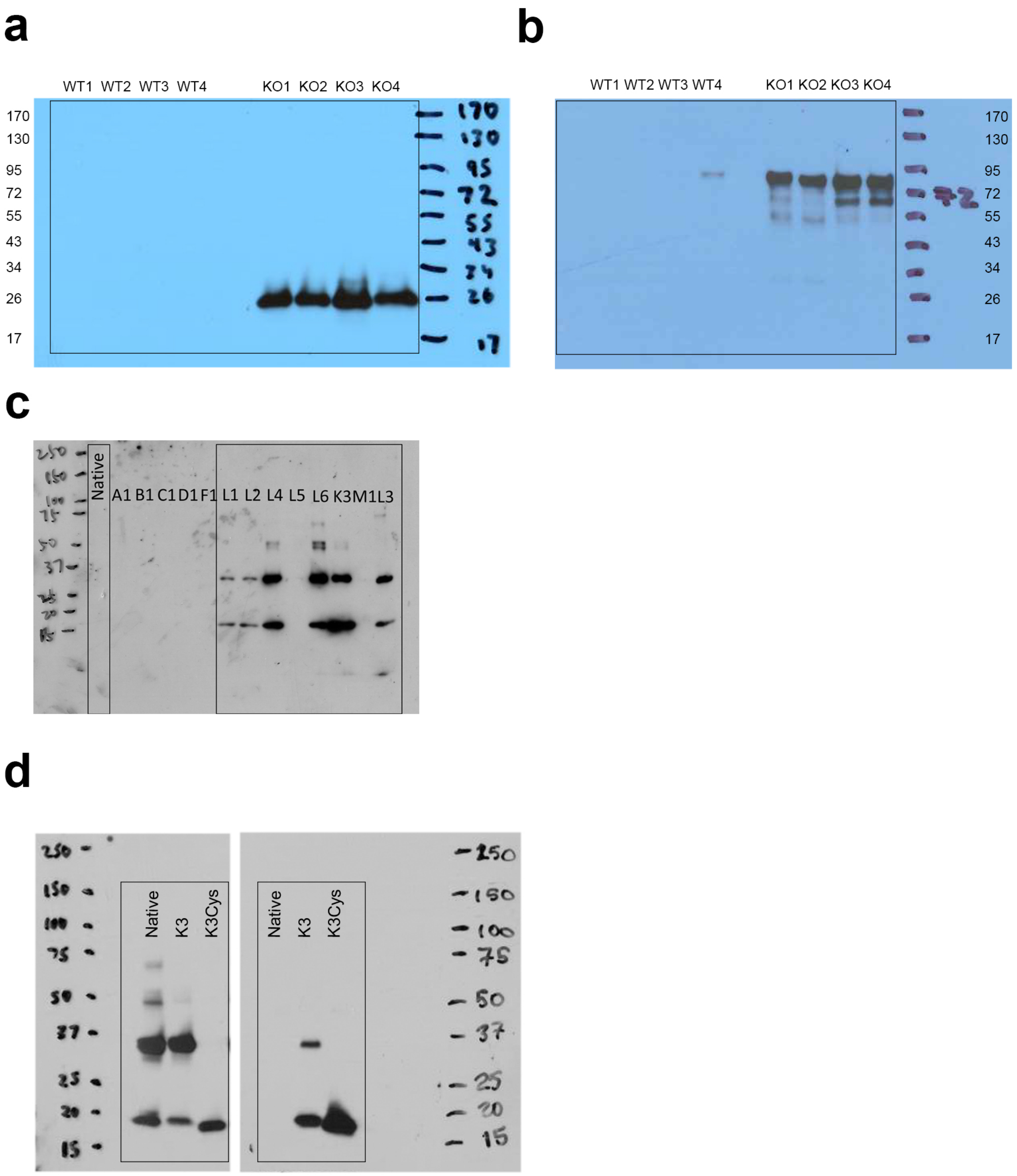

Supplementary Figure 6 | Uncropped western blots corresponding to Figure 1c (**a**), 1d (**b**), 3a (**c**), 3b (**d**)

Supplementary Table 1

Mutants	Mutation sites
A1	K15S
A2	K15S/R140Q/K142Q
A3	R43Q
B1	K98Q/H118F/H165N
B2	K15S/K46E/H118F/H165N
B3	K46E/H165N
B4	K46E/K74D
B5	K46E/K62G/K98Q/H118F/K149Q/H165N
C1	K50T/R72Q/R130Q/K149Q/K157Q
C3	K15S/K50T/R72Q/R130Q/K157Q
C5	R72Q/K157Q
D1	K73D/K74D/K75G
D2	K62G
F1	K15S/K46E/K59Q
F2	K46E/K59Q
F4	K15S/K46E/K149Q
F5	K15S/K46E/K149Q/H165N
G1	K15S/R43Q/K149Q
G3	K157Q
H1	H165N
H2	K15S/K98Q/H118F/R130Q/K149Q/H165N
H3	H118F/K149Q/H165N
H5	K15S/K98Q/H165N
I1	K15S/K73D/K74D/K75G/R130Q
I3	K62G/K73D/K74D/K75G/K98Q
I5	K15S/K62G/K73D/K74D/K75G/R130Q
K1	K15S/K46E/K59Q/K98Q/H118F/R130Q/K149Q/H165N
K2	K15S/K73D/K74D/K75G/K98Q/H118F/R130Q/K149Q/H165N
K3	K15S/K46E/K73D/K74D/K75G/K98Q/H118F/R130Q/K149Q/H165N
K3Cys	K15S/K46E/K73D/K74D/K75G/C87S/K98Q/H118F/R130Q/K149Q/H165N
K5	K15S/K46E/K98Q/H118F/R130Q/K149Q/H165N
K6	K15S/K46E/K50T/K62G/R72A/K73D/K74D/K75G/K98Q/H118F/R130Q/K149Q/H165N
K7	K15S/K46E/R72A/K73D/K74D/K75G/K98Q/H118F/R130Q/K149Q/H165N
K8	K15S/R43A/K46E/K73D/K74D/K75G/K98Q/H118F/R130Q/K149Q/K157A/H165N
L1	K62G/K73D/K74D/K75G
L2	K59A/K62G/K73D/K74D/K75G/N164D/H165A
L3	K59A/K62G/K73D/K74D/K75G/K98A
L4	K46E/K59A/K62G/K73D/K74D/K75G/K98A/Q128E/N129S/R130A
L5	K46E/K50A/K59A/K62G/K73D/K74D/K75G/K98A/Q128E/N129S/R130A
L6	K46E/K59A/K62G/K73D/K74D/K75G/K98A/N114D/Q128E/N129S/R130A
L7	K46E/K50A/K59A/K62G/K73D/K74D/K75G/K98A/N114D/Q128E/N129S/R130A
L8	K15S/K46E/K50A/K59A/K62G/K73D/K74D/K75G/K98A/Q128E/N129S/R130A/K149A
L9	K46E/K50A/K59A/K62G/K73D/K74D/K75G/K98A/Q128E/N129S/R130A/K149A
L10	K15S/K46E/K50A/K59A/K62G/K73D/K74D/K75G/K98A/Q128E/N129S/R130A
L11	K15S/K46E/K50A/K59A/K62G/K73D/K74D/K75G/K98A/N114D/N116D/Q117E/H118F/Q128E/N129S/R130A
M1	R43A/R140A/K142A/K57A
M2	R140A/K142A/K57A
